# Levels and potential health risk of heavy metals in marketed vegetables in Zhejiang, China

**DOI:** 10.1038/srep20317

**Published:** 2016-02-02

**Authors:** Xiao-Dong Pan, Ping-Gu Wu, Xian-Gen Jiang

**Affiliations:** 1 Zhejiang Provincial Center for Disease Control and Prevention, Hangzhou, China

## Abstract

The present study analyzed 5785 vegetables for concentrations of As, Cd, Cr, Pb, Ni and Hg, and estimated the health risk to local consumers by deterministic (point estimates) approaches. Levels of elements varied in different vegetables. Average levels of As, Cd, Cr, Ni, Hg and Pb were 0.013, 0.017, 0.057, 0.002, 0.094 and 0.034 mg/kg (fresh weight), respectively. The samples with 0.25% for Cd and 1.56% for Pb were exceeding the maximum allowable concentrations (MACs) set by the Chinese Health Ministry. No obvious regular geographical distribution for these metals in vegetables was found in areas of Zhejiang, China. The mean and 97.5 percentile levels of heavy metal and metalloid were used to present the mean and high exposure assessment. The health indices (HIs) were less than the threshold of 1 both in mean and high exposure assessment. It indicates that for the general people there is very low health risk to As, Cd, Cr, Pb, Ni and Hg by vegetable intake.

Vegetables play important roles in human nutrition and health, particularly as sources of vitamin C, thiamine, niacin, pyridoxine, folic acid, minerals, and dietary fiber^1^. China not only has the highest vegetable production with 16 million ton in 2012 (derived from FAO DATA STATS), but also owns large amounts of consumers in the world. So, the Chinese government and its consumers pay close attention to the pollution problem.

Heavy metal in vegetables is of growing concerns since some soils and irrigation waters are demonstrated to be polluted^2^. Vegetables easily take up heavy metals and accumulate them in their edible parts^2^. Once vegetables containing high levels of heavy metals are consumed by human, such metals can cause several clinical and physiological problems^3^,^4^.

Heavy metals such as lead (Pb), cadmium (Cd), mercury (Hg) and chromium (Cr) generally refer to metals having densities greater than 5 g/cm^3^ 5. Metalloids such as arsenic (As) often fall into the heavy metal category due to similarities in chemical properties and environmental behavior^6^. When heavy metals are dispersed into water, soil and air, they could be bio-accumulated by the crops^7^,^8^. For example, Luo *et al*.^9^ reported that high Pb level of 0.38 mg/kg in lettuce and Cd of 0.79 mg/kg in broccoli were found due to the soil contamination in an e-waste processing site of China. Gupta *et al*.^10^ observed the 17.79 mg/kg for Cd, 57. 63 g/kg for Pb in radish collected from wastewater irrigated suburban area in Titagarh, India.

Zhejiang province located in the southeast of China is a rapid developing region with a high population density. Some studies have reported heavy metal pollution in soils^6^,^7^,^11^. Therefore, it is reasonable to hypothesize that there are high heavy metal levels in vegetables and potential health risk to consumers.

In a recent study, Huang *et al*.^12^ investigated the concentrations of As, Cd, Hg and Pb in vegetables and evaluate the health risk to local consumers in Zhejiang province of China. In that study, only 343 vegetable samples of 11 species were analyzed and calculated for exposure assessment. The data revealed that at the mean exposure level all health indices (HIs) were less than the threshold of 1. The HIs of Hg and Cd at high exposure were 1.01 and 1.85. However, it should be noted that the few collected samples may lead to the uncertainty of assessment.

In the current study, we aim to analysis the levels of heavy metal and metalloid in vegetables from Zhejiang of China and evaluate the health risk to local inhabitants. We report 5785 vegetable samples of 28 species for levels of As, Cd, Cr, Pb, Ni and Hg. The health risk to local inhabitants is evaluated by estimated exposure. In addition, the detailed spatial distribution of these heavy metals in Zhejiang province is also investigated for clear understanding. Out data may provide some insight into heavy metal accumulation in vegetables and serve as a basis for comparison to other regions both in China and worldwide.

## Results and discussion

### The whole levels of metals and metalloid

Total 5785 vegetables of 28 species from Zhejiang were analyzed in this study. As shown in Table 1, the average levels of As, Cd, Cr, Ni, Hg and Pb were 0.013, 0.017, 0.057, 0.002, 0.094 and 0.034 mg/kg (fresh weight) respectively. The maximum allowable concentrations (MAC) of As, Cd, Cr, Hg and Pb in vegetables in China were 0.7, 0.2, 0.5, 0.02 and 0.2 mg/kg. Except for Cd and Pb, all mean levels of tested metals were lower than the MAC. The percentage of sample exceeding the MAC is 0.25% for Cd, 1.56% for Pb. The levels of Ni and Cr in vegetables were less than 0.08 mg/kg, which is similar with recent report^13^.

Our previous study^12^ found the average levels of As (0.009 mg/kg), Cd (0.015 mg/kg), Hg (0.003 mg/kg), and Pb (0.022 mg/kg). The present data are similar with those for As, Cd, Hg, but for Pb it is higher in this study. Furthermore, concentrations of Cr and Ni were also analyzed and estimated for their potential health risk. Our data were similar to those found in non-specific zone (not polluted)^14^,^15^. In some potential polluted areas, average levels of heavy metals, such Cd or Pb were more than 0.2 mg/kg in vegetables^16^,^17^,^18^.

### Detailed levels of metals and metalloid in vegetables

**Figure 1:**
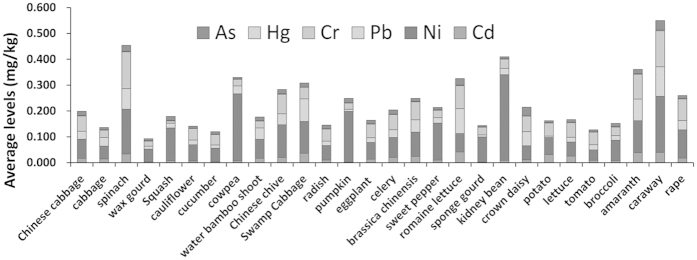
The concentration of heavy metals in 28 species of vegetables (fresh weight).

Different vegetables may accumulate different heavy metals, and the absorption ability varies in different biological species due to their diverse physiological character. The detailed levels of these metals in vegetables of 28 species can be found in Table S1. As shown in Fig. 1, *Coriandrum sativum* L. (coriander) contained the highest total mean levels of selected 6 heavy metals (0.550 mg/kg). In contrast, *Cucumis sativus* L. (cucumber) had the lowest total mean levels (0.120 mg/kg). The highest average levels of As and Pb were both found in Coriander with 0.037 and 0.114 mg/kg. The highest average levels of Cd, Cr, Ni, and Hg were in *Chicorium endiva* L. (romaine lettuce) with 0.041 mg/kg, *Spinacia oleracea* L. (spinach) with 0.142 mg/kg, *Phaseolus vulgaris* L. (kidney bean) with 0.331 mg/kg, and *Ipomoea aquatica* Forssk (swamp cabbage) with 0.0034 m/kg, respectively.

*Brassica campestris* L. (pakchoi) showed average levels of As and Cd with 0.012 and 0.017 mg/kg which were lower than previous study^12^. But mean level of Pb with 0.079 mg/kg in spinach was higher than that previous value of 0.056 mg/kg. It is hard to explain the differences between two studies. Some non-laboratory factors, such as reason, farming type and sampling areas may lead to the data diversity. Notwithstanding, data from large sampling number is more reliable than small one considering the variety in different vegetables. Similarly, other studies also revealed the high Pb in Spinach^19^.

The differences of the metal contents in these vegetables depend on the physical and chemical nature of the soil or water and absorption capacity of each metal by the plant^19^, which is altered by various factors like environmental and human interference, and the nature of the plant. The magnitude of heavy metal deposition on vegetable surfaces varied with morpho-physiological nature of the vegetables^20^. AI Jassir *et al*.^21^ have shown that unwashed leafy vegetables sold on roadside of Riyadh city, Saudi Arabia had higher levels of heavy metals as compared to washed leafy vegetables. Demirezen and Aksoy^22^ have reported higher concentrations of Pb, Cd and Cu in *Abelmoschus esculentus* collected from urban areas of Kayseri, Turkey as compared to those from rural areas. The partitioning of heavy metals is well known with accumulation of greater concentrations in the edible portions of leafy or root crops than the storage organs or fruits^23^,^24^.

### Spatial distribution of heavy metals and metalloid

**Figure 2:**
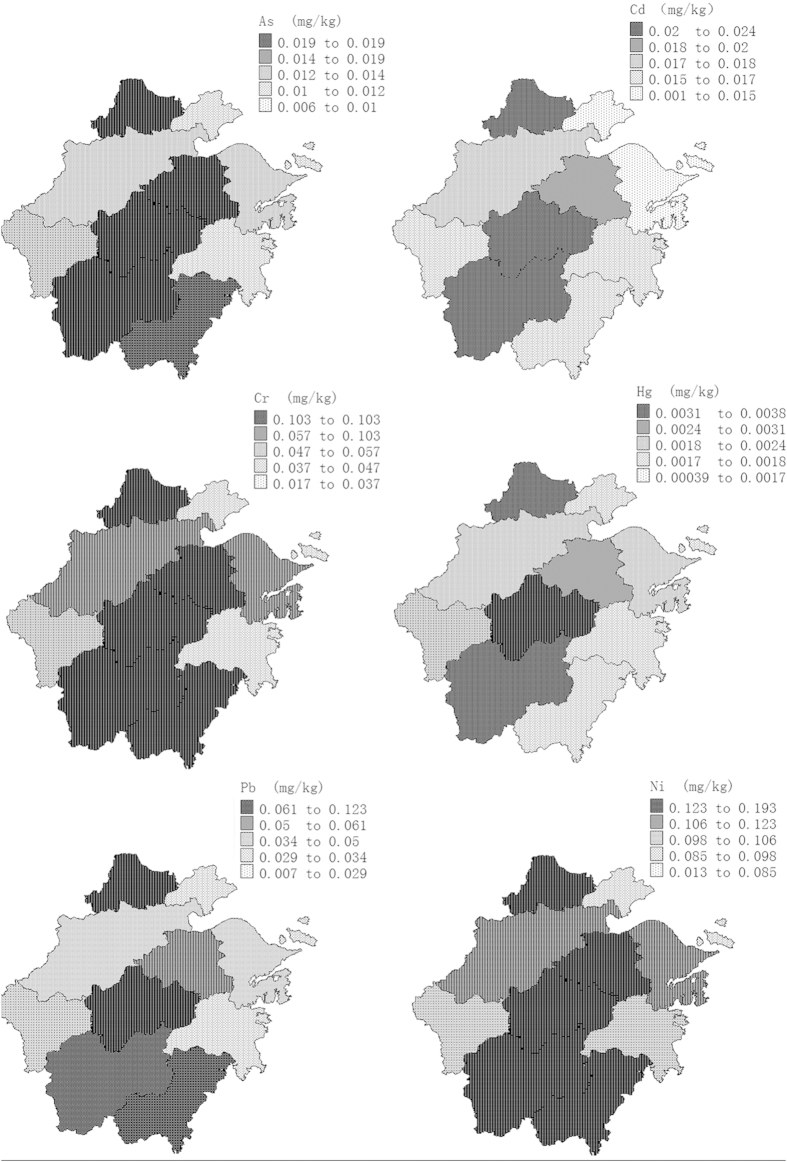
The geographical spatial distribution of the As, Cd, Cr, Hg, Pb and Ni in vegetables from Zhejiang province, China (drawn by software of MapGIS K9 SP2).

The spatial distribution of these metals and metalloid in vegetables from Zhejiang province was shown in Fig. 2. It was drawn by the software of MapGIS K9 SP2 free trial edition, and different metal and metalloid levels were marked with different color in selected areas. The different concentration ranges of metals and metalloid were classified for comparing among 11 areas. In a whole, the highest average levels of As, Cd, Cr, Pb, Ni and Hg were all found in the middle area. Furthermore, the second highest levels of As, Cr, Pb and Ni were observed in the south zones of Zhejiang. However, no obvious regular distribution for these elements in vegetables was revealed in areas of Zhejiang, China.

The sources of heavy metal pollution in vegetables are diverse. It can occur due to factors including irrigation with contaminated water, the addition of fertilizers and metal-based pesticides, industrial emissions, transportation, harvesting process, storage and/or sale. The middle of Zhejiang is the main manufacturing location for metal-origin products. So, it may have higher potential for heavy metals contamination than other region in Zhejiang.

### Exposure assessment

Based on the data of food consumption survey^25^, the estimated vegetable intake is 273.3 g/day per person. The average and P97.5 levels of heavy metal are respectively used to present the common and high exposure. Table 2 shows the estimated exposure to general population in vegetables from Zhejiang province and the health hazard index. The mean daily intakes of As, Cd, Cr, Hg, Pb and Ni by vegetables were 0.063, 0.083, 0.278, 0.010, 0.459 and 0.166 μg/kg bw/day, respectively. The high exposure of As, Cd, Cr, Hg, Pb and Ni were 0.293, 0.396, 1.221, 0.040, 2.002 and 0.830 μg/kg bw/day. Comparing with previous study of Huang *et al*.^12^, the mean exposure of As, Cd and Pb are higher in this study, but the high exposures of above metals were lower. It may be caused by the increased sample numbers. Larger number of samples probably leads to the lower dispersed degree of analyzed values.

Our mean exposure data of As and Pb were lower than those report in Turkey^22^,^26^, but little higher than in Greece and Egypt^27^,^28^. To estimate the health risk, the health hazard indices (HIs) were calculated by dividing daily intake of heavy metals by their reference doses. HI is usually adopted to assess the health risk of hazard materials in foods^29^,^30^. An HI more than 1 is considered as not safe for human health^31^. As shown in Table 2, HIs at the mean level were all less than 1. The mean exposure of intake just accounted for no more than 11% of the reference doses. It indicates that there is low health risk to common heavy metal exposure by intake of vegetables. Meanwhile, for the high exposure (P97.5 level), HIs of all tested metals were also less than 1. The HIs of Cr near to zero are the lowest, which may be ascribed to its higher tolerance dose.

The results showed that Cd and Pb were the major component contributing to HIs. Other reports also found relatively high Cd level in vegetables in Nanning, China^32^ and in Boolaroo, Australia^33^. In our study, the main contributors for Cd were romaine lettuce (0.041 mg/kg), coriander (0.040 mg/kg), *Amaranthus tricolor* L. (amaranth, 0.039 mg/kg) and swamp cabbage (0.036 mg/kg). For Pb, they were coriander (0.114 mg/kg), romaine lettuce (0.096 mg/kg), swamp cabbage (0.087 mg/kg), amaranth (0.084 mg/kg) and spinach (0.079 mg/kg) (Table S1).

### Uncertainty analysis

Deterministic approaches used here are the norm in chemical risk assessment, for instance to determine whether any risk may arise from consumption of a single food containing heavy metal. Although, using bioaccessible metals concentrations^34^ to conduct human health risk evaluations is considered to be the most reliable and accurate method^35^, it is hard to perform actually. Animal experiments to quantify the bioaccessible metal levels are costly, whereas *in vitro* digestion models are not suitable for estimating the complicated dermal contact.

In this study, the total ingested metal levels were considered as the real absorption values and taken to estimate the health risks. Thus, the health risk assessments in this study could be somewhat overestimated. Furthermore, detailed consumption data of every tested vegetable were not obtained here. We just adopted the sum of vegetable consumption data to calculate exposure values, which may lead to the uncertainty of the estimates.

### In conclusion

This study revealed the different levels of As, Cd, Cr, Ni, Hg and Pb in 5785 vegetable samples of 28 species. Comparing with the MAC of China, only 0.25% samples for Cd and 1.56% for Pb were out of range. No regular geographical distribution for these metals in vegetables was found. After the dietary exposure assessment, we conclude that that there is low health risk to As, Cd, Hg and Pb for general people in Zhejiang, China.

However, in terms of uncertainty in our assessment, further specific and detailed exposure estimates need to be performed in targeted area. In addition, the regular monitoring of heavy metals in vegetables is still necessary in this area to ensure the dietary safety.

## Materials and Methods

### Vegetable Consumption data

The vegetable consumption data used in this report was extracted from the food consumption survey conducted in 2008 by the Zhejiang Food and Drug Administration of China^25^. The representative sample of participants included 9798 people, who were questioned twice about their last 24-h consumption. The selection of interviewed people and the moment of the interview were chosen in order to obtain a representative consumption profile of the population over 1 year.

### Sampling and sample preparation

**Figure 3:**
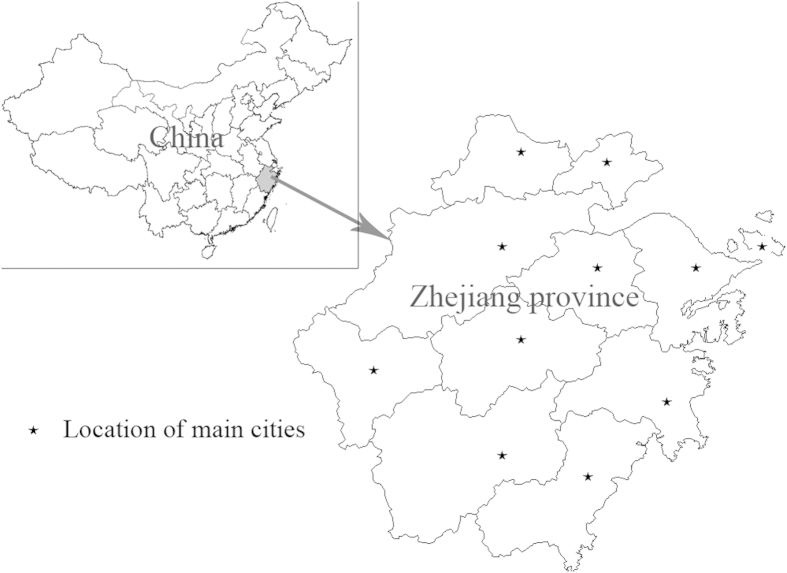
The map of the sampling locations in Zhejiang province (drawn by software of MapGIS K9 SP2).

Total 5785 vegetable samples of 28 species were collected in Mar. to Oct. 2014, from Zhejiang, an eastern coastal province of China, where the latitudes range from 27° 09′ to 31° 11′ N, and the longitudes from 118° 02′ to 122° 57′ E. There are 11 cities in Zhejiang as shown in Fig. 3. All of simple maps in this study were created by software of MapGIS K9 SP2 free trial edition (Zondy Cyber Comp., China). The vegetable and sampling number were: *Brassica rapa* subspec. *pekinensis* (Chinese cabbage, 375), *Brassica oleracea* L. var. *capitata* (cabbage, 106), *Spinacia oleracea* L. (spinach, 228), *Benincasa hispida* (Thunb.) Cogn. (wax gourd, 72), *Cucurbita pepo* L. (zucchini squash, 70), *Brassica oleracea* L. (cauliflower, 162), *Cucumis sativus* L. (cucumber, 370), *Vigna sesquipedalis* Fruw. (cowpea, 232), *Zizania caduciflora* L. (water bamboo shoot, 92), *Allium tuberosum* Rottl. ex Spreng (Chinese chive, 254), *Ipomoea aquatica* Forssk (swamp cabbage, 88), *Raphanus sativus* L. (radish, 516), *Cucurbita moschata* Duch. (pumpkin, 42), *Solanum melongena* L. (eggplant, 478), *Apium graveolens* L. (celery, 459), *Brassica chinensis* var. *chinensis* (brassica chinensis, 762), *Capsicum annuum* L. (sweet pepper, 208), *Chicorium endiva* L. (romaine lettuce, 106), *Luffa cylindrica* L. (sponge gourd, 58), *Phaseolus vulgaris* L. (kidney bean, 100), *Chryanthemum coronarium* L. (crown daisy, 92), *Solanum tuberosum* L. (potato, 58), *Lactuca sativa* L. (lettuce, 194), *Lycopersicon esculentum* Mill. (tomato, 355), *Brassica oleracea* var. *italica* L. (broccoli, 52), *Amaranthus tricolor* L. (amaranth, 102), *Coriandrum sativum* L. (coriander, 96), and *Brassica campestris* L. (pakchoi, 64). All the samples were stored at 4 °C and immediately analyzed within 24 h.

### Chemical analysis

The concentrations of As, Cd, Cr, Pb, Ni and Hg were tested as described by Huang *et al*.^12^ Briefly, the samples were digested as follows: a 5–10 g sample was added to a 100 mL round-bottom flask. Then, 10 mL concentrated nitric acid was mixed to the sample and heated for 220 °C. Hydrogen peroxide was periodically added with 1 ml until the digestion step was complete, i.e., a clear solution was reached. Finally, we diluted the solution up to a 50 mL in the volumetric flask with distilled water. The solution was analysis by Thermo SOLAAR model iCE3000 atomic absorption spectrometry (AAS) with a graphite furnace for Cd Cr, Pb and Ni, and hydride generation-atomic fluorescence (HG-AFS 9230, Jitian Co., Beijing, China) for As and Hg. Maximum allowable concentrations of contaminants in foods in China are based on the established limits by Chinese health ministry^36^,^37^.

### Validation of analytical method

The accuracy of the analytical procedures was verified by analysis of appropriate certificated reference materials (CRMs) using the same digestion and analytical methods. Two CRMs (Table 3) were purchased from National Research Center for Certified Reference Materials, China (NRCCRM). Quantitative results (within 10% of the certified value) were obtained for each metal in each CRM. Recoveries were ranged between 90.9–102.7%. Limits of Detection (LODs) were defined as 3 times the standard deviation of 10 runs of blank measurements. LODs of As, Cd, Cr, Ni, Hg and Pb were 0.005, 0.001, 0.005, 0.004, 0.005 and 0.005 mg/kg respectively.

### Exposure estimates

The data used for exposure estimates were according to the recommendation of the report Reliable Evaluation of Low-Level Contaminations of Food issued by WHO after the 2^nd^ GEMS/Food-EURO Workshop 1995^38^. Thus, a value of ^1^/_2_ LOD was assigned to all results below the LOD, where the proportion of <LOD results is not >60%. The provisional tolerable weekly intake (PTWI) of As is 21 μg/kg bw (equivalent to 3 μg/kg bw/day) according to JECFA^39^. PTMI of Cd is 25 μg/kg bw on a monthly basis (0.8 μg/kg bw/day) according to JECFA^40^. Rfd of Cr (1500 μg/kg bw/day) and Ni (20 μg/kg bw/day) is based on US EPA and WHO^41^,^42^. PTWI of Hg is 1 μg/kg bw per week according to JECFA^39^. Reference value of 1.5μg/kg bw/day for Pb is based on cardio-vascular effects according to EFSA^43^

Deterministic (point estimates) approaches were adopted here for quantification of the health risk. Exposure from vegetable was obtained by combining its consumption data and the heavy metal concentrations of the specific item and then dividing by body weight. The average body weight in this study was considered as 55.9 g^14^. The mean and 95th percentile of the daily exposure levels were used to represent the dietary exposure for average and high consumers, respectively^44^. The health risk index was calculated by dividing daily intake of heavy metals by their safe limits^32^.

## Additional Information

**How to cite this article**: Pan, X.-D. *et al*. Levels and potential health risk of heavy metals in marketed vegetables in Zhejiang, China. *Sci. Rep*. **6**, 20317; doi: 10.1038/srep20317 (2016).

## Supplementary Material

Supplementary Information

## Figures and Tables

**Table 1:** The concentration of heavy metals in vegetables from Zhejiang province (mg/kg, fresh weight).

**Element**	**n**	**Mean**^a^	**P97.5**^a^	**Range**	**MAC**^b^	**No. of > MAC**
As	1115	0.013	0.060	<LOD–0.331	0.7	0
Cd	1196	0.017	0.081	<LOD–0.340	0.2	3
Cr	769	0.057	0.250	<LOD–0.463	0.5	0
Hg	999	0.0020	0.0081	<LOD–0.0159	0.02	0
Ni	768	0.094	0.410	<LOD–1.900	–	–
Pb	1155	0.034	0.170	<LOD–0.661	0.2	18

^a^Target analytes with concentrations lower than LOD were treated as one-half of LOD when calculating the mean values.

^b^MAC, the maximum allowable concentration set by Chinese government.

**Table 2:** Estimated exposure of As, Cd, Cr, Hg, Pb and Ni to general population in vegetables from Zhejiang province and the health hazard index.

**Element**	**Safe value μg/kg bw/day**	**Daily intake assessment μg/kg bw/day**		**Health hazard index (HI)**	
		**Mean**	**P97.5**	**Mean**	**P97.5**
As	3.0	0.063	0.293	0.02	0.10
Cd	0.8	0.083	0.396	0.10	0.49
Cr	1500	0.278	1.221	0.00	0.00
Hg	0.14	0.010	0.040	0.07	0.28
Ni	20	0.459	2.002	0.02	0.10
Pb	1.5	0.166	0.830	0.11	0.55

**Table 3:** Determination of certified materials of vegetables (*n* = 6, fresh weight).

	**GBW10015 Spinach**			**GBW10014 Cabbage**		
	**Certified mg/kg**	**Measured mg/kg**	**Recovery (%)**	**Certified mg/kg**	**Measured mg/kg**	**Recovery (%)**
As	0.23 ± 0.03	0.20 ± 0.02	91.3	0.062 ± 0.014	0.06 ± 0.012	96.8
Cd	0.15 ± 0.025	0.16 ± 0.029	106.7	0.035 ± 0.006	0.034 ± 0.008	97.1
Cr	1.4 ± 0.2	1.2 ± 0.3	92.9	1.8 ± 0.3	1.7 ± 0.4	94.4
Hg	0.02 ± 0.003	0.02 ± 0.004	100.0	0.011 ± 0.002	0.010 ± 0.003	90.9
Ni	0.92 ± 0.12	0.94 ± 0.11	102.2	0.93 ± 0.10	0.95 ± 0.08	102.2
Pb	11.1 ± 0.9	11.4 ± 0.8	102.7	0.19 ± 0.03	0.18 ± 0.04	94.7
