# Supplementary material for: Levels and potential health risk of heavy metals in marketed vegetables in Zhejiang, China
Source: Sci Rep. 2016 Feb 2;6:20317. doi: 10.1038/srep20317 (PMC4735810; doi:10.1038/srep20317)
Supplement: Supplementary Information [file srep20317-s1.doc]

**Levels and potential health risk of heavy metals in marketed vegetables in Zhejiang, China**

Xiao-Dong Pan, Ping-Gu Wu, Xian-Gen Jiang*

Zhejiang Provincial Center for Disease Control and Prevention, Hangzhou, China

*Corresponding author: Xian-Gen Jiang

Email: zjupanxiaodong@hotmail.com

Tel: +86 571-87115263. Fax: +86 571-87115263.

E-mail: zjupanxiaodong@hotmail.com

Address: Physical-chemistry Room No. 201, Bin-Sheng Road No. 3399, Binjiang District, Hangzhou City, 310051, Zhejiang Provincial Center for Disease Control and Prevention, China.

Table S1 The average levels of metals and metalloids in vegetables from Zhejiang province, China

| Vegetables | number  n | Levels (mg/kg, fresh weight ) | | | | | |
| --- | --- | --- | --- | --- | --- | --- | --- |
|  |  | Cd | Ni | Pb | Cr | Hg | As |
| Chinese cabbage  *Brassica rapa* subspec. *pekinensis* | 375 | 0.017 | 0.074 | 0.030 | 0.059 | 0.0020 | 0.016 |
| Cabbage  *Brassica oleracea* L. var. *capitata* | 106 | 0.015 | 0.050 | 0.034 | 0.028 | 0.0017 | 0.008 |
| Spinach  *Spinacia oleracea* L. | 228 | 0.034 | 0.173 | 0.079 | 0.142 | 0.0027 | 0.022 |
| Wax gourd  *Benincasa hispida* (Thunb.) Cogn. | 72 | 0.004 | 0.049 | 0.012 | 0.019 | 0.0022 | 0.007 |
| Zucchini squash  *Cucurbita pepo* L. | 70 | 0.008 | 0.126 | 0.018 | 0.009 | 0.0022 | 0.015 |
| Cauliflower  *Brassica oleracea* L. | 162 | 0.007 | 0.063 | 0.018 | 0.044 | 0.0014 | 0.007 |
| Cucumber  *Cucumis sativus* L. | 370 | 0.004 | 0.052 | 0.012 | 0.040 | 0.0015 | 0.010 |
| Cowpea  *Vigna sesquipedalis* Fruw. | 232 | 0.008 | 0.259 | 0.031 | 0.026 | 0.0017 | 0.005 |
| Water bamboo shoot  *Zizania caduciflora* L. | 92 | 0.017 | 0.074 | 0.044 | 0.027 | 0.0019 | 0.014 |
| Chinese chive  *Allium tuberosum* Rottl. ex Spreng | 254 | 0.020 | 0.127 | 0.042 | 0.076 | 0.0021 | 0.016 |
| Swamp cabbage  *Ipomoea aquatica* Forssk | 88 | 0.036 | 0.123 | 0.087 | 0.044 | 0.0034 | 0.013 |
| Radish  *Raphanus sativus* L. | 510 | 0.010 | 0.056 | 0.017 | 0.047 | 0.0019 | 0.013 |
| Pumpkin  *Cucurbita moschata* Duch. | 42 | 0.003 | 0.196 | 0.008 | 0.025 | 0.0014 | 0.016 |
| eggplant  *Solanum melongena* L. | 478 | 0.014 | 0.065 | 0.018 | 0.053 | 0.0019 | 0.013 |
| Celery  *Apium graveolens* L. | 459 | 0.021 | 0.077 | 0.030 | 0.059 | 0.0024 | 0.016 |
| Brassica chinensis  *Brassica chinensis* var. *chinensis* | 762 | 0.025 | 0.094 | 0.047 | 0.070 | 0.0023 | 0.011 |
| Sweet pepper  *Capsicum annuum* L. | 208 | 0.010 | 0.143 | 0.021 | 0.028 | 0.0024 | 0.009 |
| Romaine lettuce  *Chicorium endiva* L. | 106 | 0.041 | 0.072 | 0.096 | 0.089 | 0.0025 | 0.025 |
| Sponge gourd  *Luffa cylindrical* L. | 58 | 0.002 | 0.097 | 0.008 | 0.030 | 0.0017 | 0.004 |
| Kidney bean  *Phaseolus vulgaris* L. | 100 | 0.009 | 0.331 | 0.025 | 0.036 | 0.0019 | 0.007 |
| Crown daisy  *Chryanthemum coronarium* L. | 92 | 0.011 | 0.055 | 0.054 | 0.060 | 0.0030 | 0.032 |
| Potato  *Solanum tuberosum* L. | 58 | 0.032 | 0.066 | 0.005 | 0.051 | 0.0021 | 0.007 |
| Lettuce  *Lactuca sativa* L. | 194 | 0.025 | 0.055 | 0.018 | 0.056 | 0.0017 | 0.010 |
| Tomato  *Lycopersicon esculentum* Mill. | 355 | 0.010 | 0.040 | 0.020 | 0.049 | 0.0015 | 0.008 |
| Broccoli  *Brassica oleracea* var. *italic* L. | 52 | 0.008 | 0.079 | 0.018 | 0.033 | 0.0023 | 0.012 |
| Amaranth  *Amaranthus tricolor* L. | 102 | 0.039 | 0.124 | 0.084 | 0.096 | 0.0023 | 0.016 |
| Coriander  *Coriandrum sativum* L. | 96 | 0.040 | 0.216 | 0.114 | 0.139 | 0.0030 | 0.037 |
| Pakchoi  *Brassica campestris* L. | 64 | 0.017 | 0.110 | 0.035 | 0.084 | 0.0018 | 0.012 |
